# Using Combined Morphological, Allometric and Molecular Approaches to Identify Species of the Genus *Raillietiella* (Pentastomida)

**DOI:** 10.1371/journal.pone.0024936

**Published:** 2011-09-20

**Authors:** Crystal Kelehear, David M. Spratt, Sylvain Dubey, Gregory P. Brown, Richard Shine

**Affiliations:** 1 School of Biological Sciences, University of Sydney, Camperdown, NSW, Australia; 2 CSIRO Ecosystem Sciences, Canberra, ACT, Australia; University of Lausanne, Switzerland

## Abstract

Taxonomic studies of parasites can be severely compromised if the host species affects parasite morphology; an uncritical analysis might recognize multiple taxa simply because of phenotypically plastic responses of parasite morphology to host physiology. Pentastomids of the genus *Raillietiella* are endoparasitic crustaceans primarily infecting the respiratory system of carnivorous reptiles, but also recorded from bufonid anurans. The delineation of pentastomids at the generic level is clear, but the taxonomic status of many species is not. We collected raillietiellids from lungs of the invasive cane toad (*Rhinella marina*), the invasive Asian house gecko (*Hemidactylus frenatus*), and a native tree frog (*Litoria caerulea*) in tropical Australia, and employed a combination of genetic analyses, and traditional and novel morphological methods to clarify their identity. Conventional analyses of parasite morphology (which focus on raw values of morphological traits) revealed two discrete clusters in terms of pentastome hook size, implying two different species of pentastomes: one from toads and a tree frog (*Raillietiella indica*) and another from lizards (*Raillietiella frenatu*s). However, these clusters disappeared in allometric analyses that took pentastome body size into account, suggesting that only a single pentastome taxon may be involved. Our molecular data revealed no genetic differences between parasites in toads *versus* lizards, confirming that there was only one species: *R. frenatus*. This pentastome (previously known only from lizards) clearly is also capable of maturing in anurans. Our analyses show that the morphological features used in pentastomid taxonomy change as the parasite transitions through developmental stages in the definitive host. To facilitate valid descriptions of new species of pentastomes, future taxonomic work should include both morphological measurements (incorporating quantitative measures of body size and hook bluntness) and molecular data.

## Introduction

Delineating species in parasitic organisms can pose major logistical challenges, especially if the traits used for species definition and recognition are affected by host physiology. In this paper we explore an example of such a system, involving pentastomids. These endoparasites are believed to be the oldest metazoan parasites known to science; prehistoric larvae closely resembling extant primary larvae appeared in the fossil record ∼100 million years prior to the vertebrates they now parasitize [1]. As adults, pentastomids inhabit the respiratory system of vertebrates, maturing primarily in carnivorous reptiles (90% of pentastomid species mature in snakes, lizards, crocodiles and chelonians), but also in toads, birds (seabirds and vultures), and mammals (canines, felines, reindeer, sugar gliders and humans). Adult pentastomes feed primarily on blood from host capillary beds and can cause severe pathology, sometimes resulting in death [2].

The class Pentastomida comprises two orders: Cephalobaenida and Porocephalida. Within the order Cephalobaenida, the family Cephalobaenidae contains the largest pentastome genus: *Raillietiella*, comprised of ∼39 species. Raillietiellids are small pentastomids, generally <25 mm, that mature primarily in the lungs of reptiles, most commonly in small lizards. Like other pentastomids, raillietiellids have piercing mouthparts surrounded by two pairs of compound hooks that embed into the lung to facilitate feeding on whole blood. They reproduce sexually and females lay large numbers of eggs that pass up the trachea, are swallowed, and pass out to the environment with feces. The life cycle is unknown for most species but it seems that the eggs often are ingested by a coprophagous insect intermediate host (such as a cockroach) before developing into infective larvae. When the insect is consumed by an appropriate definitive host, the larvae burrow out of the stomach and migrate to establish infection in the host lungs [3].

Toads are the only amphibians known to act as definitive hosts for raillietiellids. Three species have been reported to mature exclusively in the lungs of toads, *Raillietiella bufonis* in Puerto Rican *Peltophryne lemur* (formerly *Bufo lemur*) [4]; *Raillietiella indica* in Hawaiian *Rhinella marina* (formerly *Bufo marinus*) [5]; and *Raillietiella rileyi* in Malaysian *Duttaphrynus melanostictus* (formerly *Bufo melanostictus*) [6]. Further to these three well-established host-parasite associations, *Raillietiella freitasi*, generally known from lizards, has been reported in Brazilian *Rhinella schneideri* (formerly *Bufo paracnemis*) [7], and a single unidentified raillietiellid was recovered from the lungs of Nigerian *Amietophrynus regularis* (formerly *Bufo regularis*) [8]. There are no recorded amphibian hosts for raillietiellids within Australia, but three raillietiellids mature in reptiles: *Raillietiella amphiboluri* infects a native dragon, *Pogona barbata* [9]; *Raillietiella scincoides* infects a native skink, *Tiliqua scincoides* [10] and a native gecko, *Nephrurus laevissimus* [11], and *Raillietiella frenatus* infects an introduced gecko, *Hemidactylus frenatus*, and a native gecko, *Gehyra australis* [12]. Mature *Raillietiella* sp. also have been reported in two Australian snake species, *Pseudechis australis* and *Pseudonaja textilis* [13].

Historically, descriptions of new species of pentastomids have been based on morphological features, with emphasis placed on pentastome body size, the number of body annuli, the morphology of the two pairs of retractile hooks, the morphology of the buccal cadre, and the morphology of the male copulatory spicules [14]. However, due to the small numbers of specimens generally examined, the method of fixation employed, the state of the preserved type or voucher specimens, and intraspecific variation in the aforementioned morphological traits, there are many misidentifications in the pentastome literature (i.e., where new species have been named in error). For example, *Raillietiella hebitihamata* was described as a “new species” from reptiles in Taiwan [15], but was later concluded to be synonymous with *Raillietiella hemidactyli* [16]. Subsequent reexamination of type specimens suggested that the raillietiellid in question was in fact *R. frenatus*, not *R. hemidactyli* [17].

Although the delineation of pentastomids at the generic level is clear, the taxonomic status of many species remains abstruse. A review of the principal characteristics of the genus *Raillietiella* [18], and of the major taxonomic groupings [19]–[22], split all recognized species into seven groups defined primarily by the hosts in which they occur and the morphology of their posterior hooks. During the course of parasite surveys in cane toads, we discovered raillietiellids with posterior hooks ranging from sharp through to blunt, with the blunt-hooked species resembling *R. frenatus* from geckos. Under the current system of taxonomic groupings [18] the raillietiellids we discovered in toads therefore spanned at least two species groups: species from small lizards with blunt posterior hooks, and species from toads. We thus conducted a study to clarify the identity of raillietiellids infecting two sympatric invasive host species in tropical Australia: the cane toad (*R. marina*) and the Asian house gecko (*H. frenatus*). We combined genetic methods with traditional morphological methods (as used by previous researchers) as well novel protocols for objectively quantifying a taxonomically important trait (hook bluntness [18]), and analyses that incorporated parasite body size to allow for possible allometries in trait morphology. These novel methods enabled us to reach conclusions about species identity that would have been impossible with the methods typically used in this field (i.e., the use of means-based morphological data only). Our results thus not only clarify the identity of the pentastomes infecting native and invasive amphibians and reptiles in tropical Australia, but also have significant methodological implications for future taxonomic studies on pentastomes.

## Results

### Molecular Results

Of the 26 specimens from *R. marina*, 11 had relatively sharp posterior hooks (resembling *R. indica*) and 15 had comparatively blunt posterior hooks (resembling *R. frenatus*). All eight specimens from the gecko *H. frenatus* were identified morphologically as *R. frenatus*. Despite the morphological determinations, only one haplotype of 617 bp (Genbank Accession Number: JF975594) was identified in this material; consequently, no genetic variation was present within these 34 samples. The two samples of *Waddycephalus* sp. isolated from two different snakes showed distinct haplotypes (K2P distance: 0.3%; number of mutations: two; Genbank Accession Numbers: JF975595, JF975596) and were genetically distinct from the *Raillietiella* samples (K2P distance: 39.5%; number of mutations between *Raillietiella* and *Waddycephalus* outgroups: 188).

### Morphological Results

**Figure 1:**
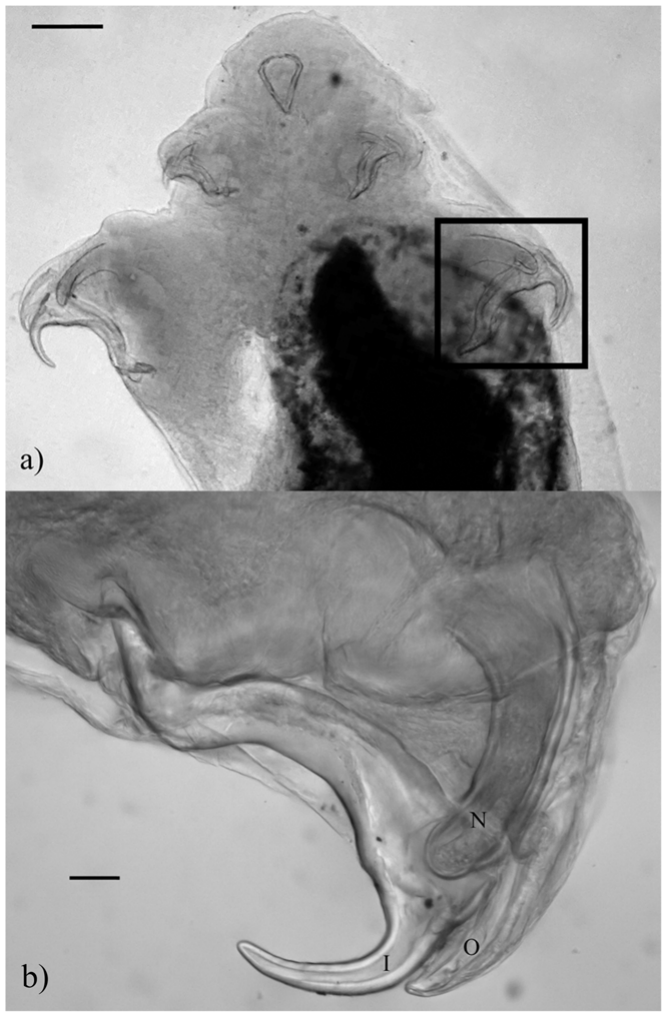
Molting *Raillietiella frenatus* that has retained the hooks of two previous instars. (a) Anterior view of molting *R. frenatus* from *Hemidactylus frenatus*, scale bar is 0.1 mm. (b) Close-up of posterior hooks from the same individual showing retained posterior hooks from two previous instars. The oldest hook is marked O, the intermediate aged hook is marked I, the newest hook is marked N. Photograph is compiled from five focus-stacked images of the same field of view, scale bar is 20 *µ*m.

One pentastomid recovered from *H. frenatus* retained the posterior pair of hooks from two previous instars (Fig. 1). The morphology of the distal extremities of the hooks changed from sharply pointed (characteristic of *R. indica)*, to more bluntly pointed (resembling an intermediate of *R. indica* and *R. frenatus*), to the very blunt-ended hook characteristic of adult *R. frenatus* (Fig. 1).

**Figure 2:**
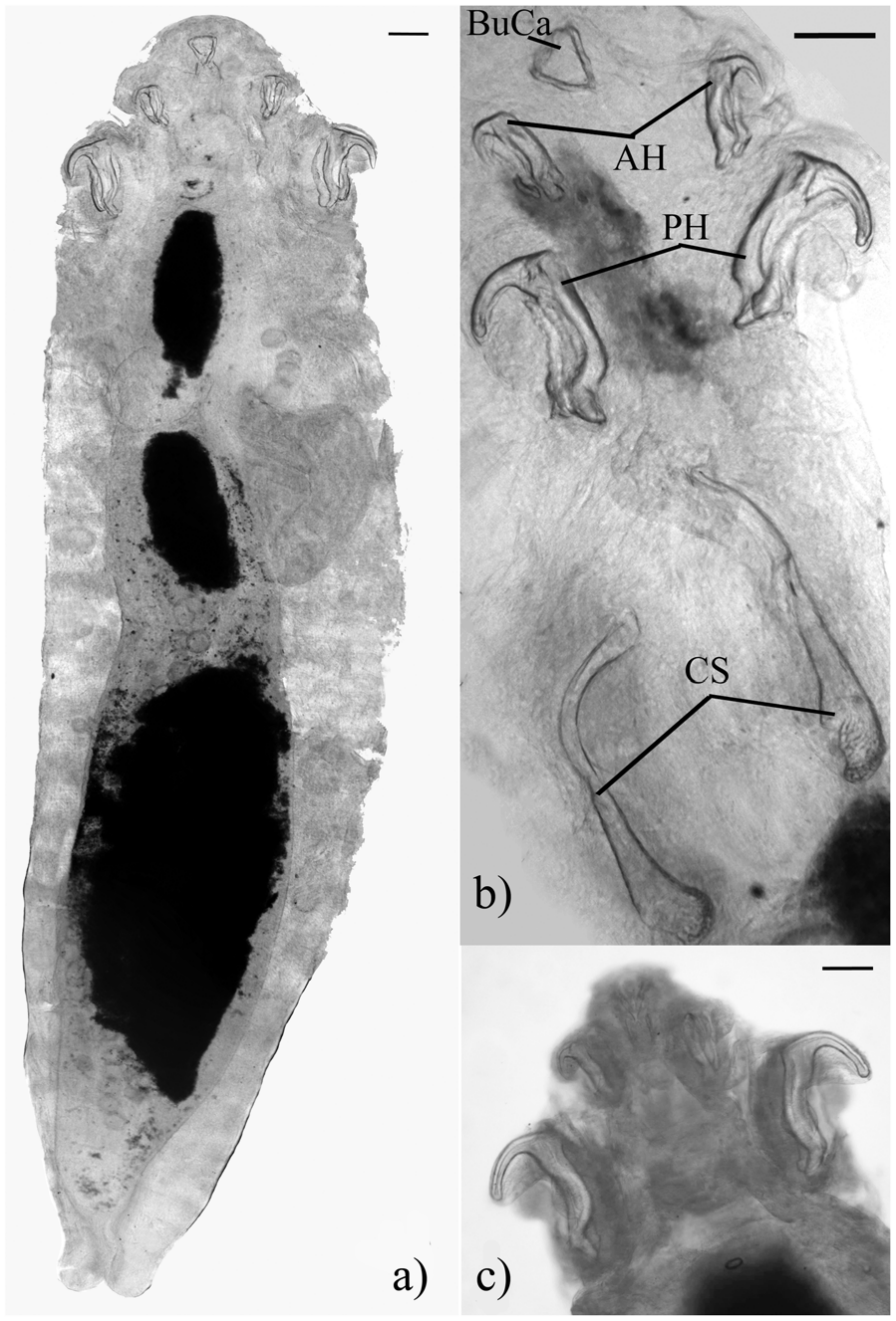
Photographs of *Raillietiella frenatus* showing key features. (a) Female *Raillietiella frenatus* (bearing morphological resemblance to *Raillietiella indica*) from *Rhinella marina* showing entire specimen. Note sharp anterior hooks and intermediately sharp posterior hooks. Photograph is complied from five images stitched longitudinally together. (b) Anterior view of male *R. frenatus* from *Hemidactylus frenatus*, note buccal cadre (BuCa), sharp anterior hooks (AH), relatively blunt posterior hooks (PH) and copulatory spicules (CS). (c) Anterior view of female *R. frenatus* from *H. frenatus*, note blunt posterior hooks. Scale bars are all 0.1 mm.

**Figure 3:**
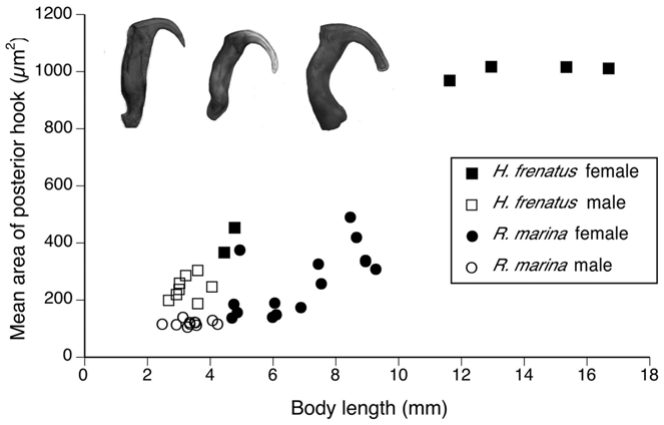
Area of posterior hook tips of *Raillietiella frenatus* graphed against pentastome body length. Small areas indicate relatively sharp hooks, large areas indicate relatively blunt hooks (see example pictures inset with sharp, intermediate and blunt hook tips).

**Figure 4:**
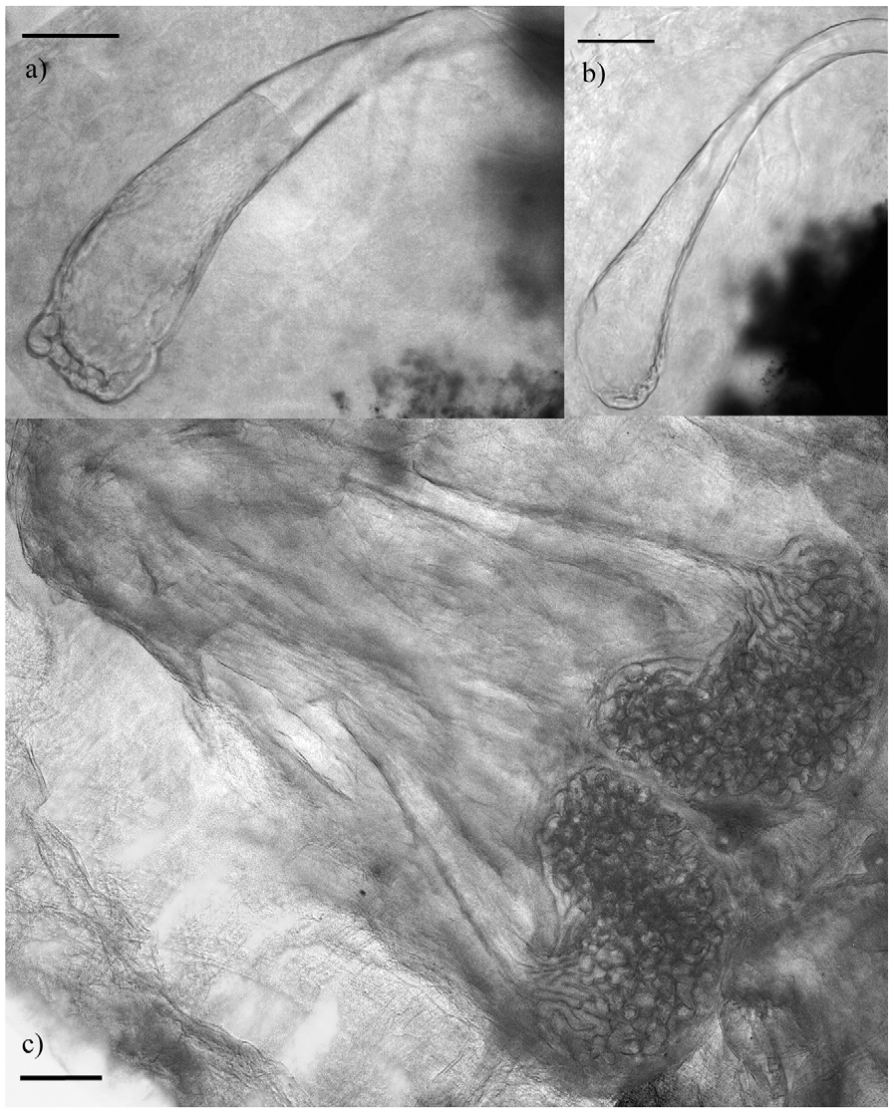
Copulatory spicules of *Raillietiella frenatus* and *Raillietiella orientalis*. (a) Copulatory spicule of male *Raillietiella frenatus* from *Rhinella marina.* Photograph is compiled from three focus-stacked images of the same field of view, scale bar is 0.05 mm. (b) Copulatory spicule of male *R. frenatus* from *Hemidactylus frenatus*. Photograph is compiled from two focus-stacked images of the same field of view, scale bar is 0.05 mm. (c) Paired copulatory spicules of male *Raillietiella orientalis* from *R. marina*. Note flared ornamented bases, scale bar is 0.1 mm.

Measurements of morphological features are presented as the range observed across all 40 specimens examined from toads and geckos. All pentastomids recovered were cylindrical to fusiform in shape (Fig. 2a). Body length ranged from 2.5 to 16.7 mm, and width from 0.5 to 2.4 mm (Table 1). All anterior hooks were sharp (Fig. 2a,b), and posterior hooks ranged from sharp (two ethanol-preserved specimens deposited in the Australian National Wildlife Collection, accession numbers W/L HC#P135, P136) to blunt (Fig. 2, Fig. 3, Table 1). AB of anterior hooks ranged from 49.1 to 139.4 *µ*m, BC of anterior hooks ranged from 93.2 to 222.6 *µ*m, and area of the anterior hook tips ranged from 65.0 to 196.6 *µ*m^2^ (Table 1). AB of posterior hooks ranged from 90.1 to 344.1 *µ*m, BC of posterior hooks ranged from 142.3 to 483.3 *µ*m, and area of posterior hook tips ranged from 105.6 to 1017.0 *µ*m^2^ (Table 1). Male copulatory spicules were club shaped with ornamented bases (Fig. 2b, 4a,b) and were 353.9–482.8 *µ*m long, and 60.1–90.2 *µ*m wide (Table 1).

**Table 1:** Morphological measurements of *Raillietiella frenatus* from *Rhinella marina* and *Hemidactylus frenatus*.

Host	#	Sex	Body length (mm)	Body width (mm)	AB anterior hook (*µ*m)	BC anterior hook (*µ*m)	Area anterior hook tip (*µ*m^2^)	AB posterior hook (*µ*m)	BC posterior hook (*µ*m)	Area posterior hook tip (*µ*m^2^)	Spicule length (*µ*m)	Spicule width (*µ*m)
*R. marina*	15	F	4.7–9.3 (6.9±0.4)	0.7–2.4 (1.5±0.1)	49.1–130.0 (92.0±6.6)	125.8–200.1 (166.1±6.9)	92.2–141.1 (110.5±3.8)	138.4–329.1 (232.1±19.9)	198.4–419.9 (308.0±19.0)	137.7–490.6 (265.7±29.3)	—	—
*R. marina*	11	M	2.5–4.2 (3.4±0.1)	0.7–1.2 (1.0±0.0)	46.6–67.7 (55.6±1.8)	93.2–118.9 (108.8±2.7)	72.8–120.3 (92.6±5.0)	90.1–113.2 (101. 9±2.0)	142.3–184.2 (168.2±4.7)	105.6–139.5 (119.4±2.7)	353.9–480.2 (442.5±11.5)	60.1–90.2 (77.7±3.0)
*H. frenatus*	6	F	4.4–16.7 (11.0±2.1)	0.9–2.0 (1.5±0.2)	60.7–139.4 (102.4±14.4)	138.9–222.6 (183.6±14.9)	87.5–196.6 (123.5±17.1)	183.3–344.1 (279.3±30.6)	261.5–483.3 (388.1±34.9)	366.6–1017.0 (805.4±125.8)	—	—
*H. frenatus*	8	M	2.7–4.1 (3.3±0.2)	0.5–1.0 (0.7±0.1)	51.0–70.9 (61.1±2.8)	100.9–121.4 (114.0±2.8)	65.0–133.4 (97.4±7.0)	124.7–140.6 (132.0±2.2)	183.4–210.3 (198.2±3.2)	188.0–304.5 (242.8±14.3)	389.1–482.8 (435.1±10.5)	64.8–80.8 (72.2±1.7)

AB = barb length, BC = overall length.

We report minimum and maximum measurements for each feature, and means ± SE in brackets.

**Figure 5:**
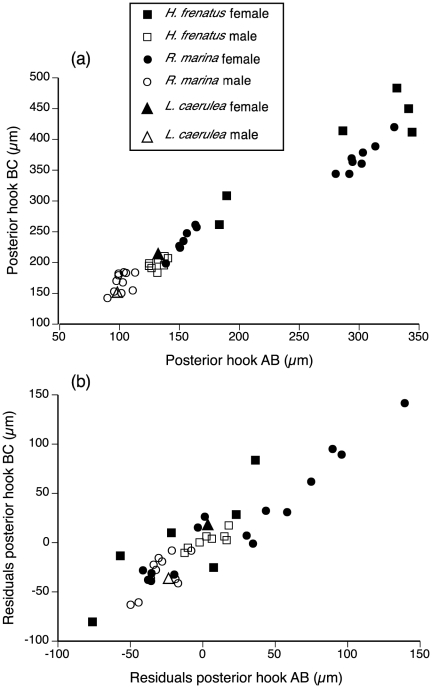
Barb length (AB) *versus* overall length (BC) of posterior hooks of *Raillietiella frenatus* from *Rhinella marina*, *Hemidactylus frenatus*, and *Litoria caerulea*. (a) Raw hook measurements uncorrected for pentastome body size, note two visible clusters. (b) Hook measurements corrected for pentastome body size (by calculating residual scores from a linear regression of hook measurements against pentastome body size), note that clusters now disappear.

In keeping with methods employed in previous studies, we first plotted AB (barb length) against BC (overall length) of posterior hooks to visualize distinct clusters indicative of separate species. All measurements from male pentastomes from *R. marina* clustered tightly whereas female pentastomes from *R. marina* formed two distinct clusters, implying two separate species (Fig. 5a). When we included body size as a covariate in the analysis, these discrete clusters disappeared (Fig. 5b). Regressions of pentastome body length against AB and BC of anterior and posterior hooks, and hook tip area (Fig. 3) were positively correlated (*P≤*0.0002 in all cases), indicating that hooks become larger and blunter with age. Length and width of copulatory spicules did not scale with body length (*F*_1,16_ = 0.16, *P* = 0.70, and *F*_1,17_ = 0.04, *P* = 0.84, respectively). Female pentastomes attained greater lengths in geckos than in cane toads, whilst male body size remained unchanged between the two host species (*F*_1,36_ = 7.88, *P* = 0.008).

**Figure 6:**
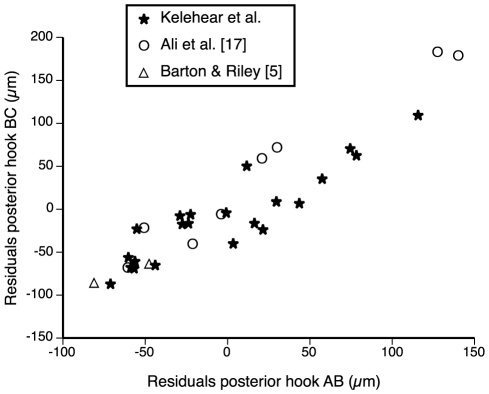
Barb length (AB) *versus* overall length (BC) of posterior hooks of *Raillietiella frenatus* from the current study compared to the literature. Kelehear *et al.* (2011, current study) measured *R. frenatus* from *Rhinella marina* and *Hemidactylus frenatus*; Ali *et al.* (1981) measured *R. frenatus* from *H. frenatus*; and Barton and Riley (2004) measured “*R. indica*” from *R. marina*. All measurements corrected for pentastome body size by calculating residual scores from a linear regression of hook measurements against pentastome body size.

AB of posterior and anterior hooks was not related to sex (*F*_1,33_ = 3.61, *P* = 0.07, and *F*_1,33_ = 1.87, *P* = 0.18, respectively), but there was a significant interaction between host species and body length (*F*_1,33_ = 8.17, *P* = 0.0007, and *F*_1,33_ = 5.10, *P* = 0.03), reflecting steeper slopes in the relationship between body length and AB of posterior and anterior hooks in *R. marina* compared to *H. frenatus*. That is, as pentastome body size increases, AB of hooks increases faster in pentastomes parasitizing toads than in those parasitizing geckos. Mean BC of posterior and anterior hooks was influenced by sex (*F*_1,36_ = 15.75, *P* = 0.0003, and *F*_1,36_ = 16.47, *P* = 0.0003 respectively) but not by host species (*F*_1,36_ = 2.37, *P* = 0.13 respectively, and *F*_1,36_ = 0.004, *P* = 0.95 respectively). BC was relatively longer in females than males, even after considering their larger body sizes (Fig. 6). Area of the posterior hook tips was not influenced by sex (*F*_1,36_ = 0.06, *P* = 0.81; Fig. 4), but this measurement was highest in pentastomes from geckos (*F*_1,36_ = 51.21, *P*<0.0001; Fig. 4), indicating that pentastomes in geckos have the bluntest posterior hooks. Area of the anterior hook tips was not influenced by sex (*F*_1,36_ = 0.08, *P* = 0.78) or host species (*F*_1,36_ = 0.05, *P* = 0.82). There was no effect of host species on spicule length (*F*_1,16_ = 0.21, *P* = 0.65) or spicule width (*F*_1,17_ = 2.03, *P* = 0.17).

Published raw data on morphology were available only for female pentastomes. The regressions included data from eight females measured by Ali et al. [17], two females measured by Barton and Riley [5], and 22 females from *R. marina*, *H. frenatus* and *L. caerulea* measured in the current study. Our pentastomes did not differ in mean length to those measured in previous studies (*F*_2,28_ = 1.57, *P* = 0.23). Across the pooled data, both AB and BC of anterior and posterior hooks were strongly positively correlated with pentastome body length (*P*<0.0001 in all cases). AB of anterior hooks was not given in Barton and Riley [5], but measurements in Ali et al. [17] did not differ from the current study (*F*_1,24_ = 0.25, *P* = 0.63). The studies did not differ significantly in terms of mean BC of anterior hooks (*F*_1,27_ = 2.56, *P* = 0.10) or mean AB of posterior hooks (*F*_1,27_ = 1.76, *P* = 0.19; Fig. 6), but mean BC of posterior hooks did vary (*F*_1,27_ = 3.24, *P* = 0.05; Fig. 6); measurements from Barton and Riley [5] were smaller than those from Ali et al. [17], but those taken in the current study were not significantly different to the measurements taken by either of the other authors (Least Squares means differences Student's *T* test).

### Incidental Findings

The unusually large pentastomes observed in two cane toads, one from Windows on the Wetlands and one from the Adelaide River, were identified as *R. orientalis* (one ethanol-preserved specimen deposited in the Australian National Wildlife Collection, accession number W/L HC#P134) on the basis of hook dimensions, and size and appearance of copulatory spicules (Fig. 4c). Female: 18 mm long, 1.4 mm wide; AB of anterior hooks 212 *µ*m; BC of anterior hooks 276 *µ*m; AB of posterior hooks 265 *µ*m; BC of posterior hooks 302 *µ*m. Males: 12, 13 mm long, 1.5, 1.7 mm wide; AB of anterior hooks 174, 186 *µ*m; BC of anterior hooks 212, 212 *µ*m; AB of posterior hooks 228, 215 *µ*m; BC of posterior hooks 249, 286 *µ*m; spicules 928, 875 and 885, 906 *µ*m long, 281, 371 and 318, 228 *µ*m wide.

The green tree frog had three pentastomes in its lungs (one female, one male, one juvenile), identified as *R. frenatus* on the basis of morphology of the male copulatory spicules and measurements of the male and female. Female: 3.32 mm long, 0.95 mm wide; AB of anterior hooks 58 *µ*m; BC of anterior hooks 136 *µ*m; area of anterior hook tips 80 *µ*m^2^; AB of posterior hooks 132 *µ*m; BC of posterior hooks 216 *µ*m; area of posterior hook tips 107 *µ*m^2^. Male: 3.00 mm long, 0.97 mm wide; AB of anterior hooks 55 *µ*m; BC of anterior hooks 100 *µ*m; area of anterior hook tips 90 *µ*m^2^; AB of posterior hooks 98 *µ*m; BC of posterior hooks 153 *µ*m; area of posterior hook tips 124 *µ*m^2^; spicules 370 *µ*m long, 66 *µ*m wide.

We were unable to obtain molecular data for these specimens and did not consider them in the above analyses.

## Discussion

Initial morphological results indicated two pentastome species infecting the lungs of the introduced cane toad (*R. indica* and *R. frenatus*), and one species (*R. frenatus*) infecting the lungs of the introduced Asian house gecko. However, there was no genetic difference between specimens of *Raillietiella* recovered from cane toads and geckos and these we now consider to be *R. frenatus*. We thus conclude that the specimens in cane toads identified morphologically as *R. indica*, on the basis of body size, hook dimensions and sharpness of the posterior hook tips, are in fact early stages of *R. frenatus*. The fortuitous finding of a specimen of *R. frenatus* (from a gecko) in which the posterior pairs of hooks had been retained from two previous instars, confirmed this conclusion. Hence, the morphology of the posterior pairs of hooks of *R. frenatus* changes markedly as the parasite transitions through different developmental stages in the definitive host.

*Raillietiella frenatus* is previously known only from lizards, and our study provides the first instances of the species maturing in amphibians (the toad *R. marina*, and the tree frog *L. caerulea*). Considering that the intermediate host for *R. frenatus* is an insect [3], the discovery of new insectivorous definitive hosts is unsurprising. Bufonids were previously the only known amphibian hosts of pentastomes. *Raillietiella indica* has been reported from bufonids previously and despite much debate (see below) it is likely that this “species” is actually an early stage of *R. frenatus*. Gedoelst [23] originally described *R. indica* on the basis of its small size. The type specimen was subsequently re-examined by Hett [24] who considered it an immature female, a conclusion upheld by Heymons [19] who postulated that the toad was an intermediate host. Hett [25] later reversed her opinion and claimed the type material was a “ripe” female, describing additional specimens from *Duttaphrynus melanostictus* and placing *R. affinis* (Bouvien, 1923), a small raillietiellid from a gecko in Java, as a synonym of *R. indica*. Subsequent reviews of pentastomid taxonomy and life histories subscribed to the view that *R. indica* was an immature stage [20], [21]. However, in the most recent assessment Ali et al. [4] concluded that *R. indica* was almost certainly a valid species founded on a mature type specimen, based on finding what they considered to be mature males of the same species in the same host species (their Fig. 1A).

*Raillietiella indica* was recently identified from Hawaiian *R. marina* based on the morphology of two females and seven males [5]. The authors speculated that *R. indica* may have either been introduced to Hawaii with an insect intermediate host or with its definitive host *D. melanostictus*, although this latter toad species (native to southern Asia) has not been reported in Hawaii [26]. Four species of Hawaiian lizards are known to be infected with *R. frenatus*: the Asian house gecko (*H. frenatus*) [27], the mourning gecko (*Lepidodactylus lugubris*) [27], the brown anole (*Anolis sagrei*) [28], and the Madagascan giant day gecko (*Phelsuma grandis*) [29]. In light of our findings, the parasite reported in Hawaiian cane toads may well be *R. frenatus*, introduced to Hawaii with its invasive host *H. frenatus*.

We examined the morphological features typically used in descriptions of new species of pentastomids, with the addition of hook area as a quantitative measure of hook bluntness, and the exception of number of body annuli. This latter feature exhibits marked interspecific overlap and intraspecific variation, and is difficult to quantify on alcohol-preserved specimens [14]. Comparing our measurements with those of alcohol-preserved specimens in the literature, the pentastomes in our study did not differ in mean length to those measured by Ali et al. [17] and Barton and Riley [5]. Our female pentastomes attained greater lengths in geckos than in cane toads, whereas male pentastomes attained similar sizes in both host species. The pentastomid *Armillifer armillatus* attains larger body sizes in puff adders (*Bitis* spp.) than it does in African pythons (*Python* spp.) [30]. Presumably this size disparity between hosts reflects differences in host physiology, perhaps involving nutrient availability and host immune responses.

Pentastome body size was strongly correlated with all measures of hook morphology, a pattern overlooked in published literature. Our graphs highlight the importance of including body size as a covariate; without consideration of body size there appear to be two clusters (and hence, two species [14]), yet after incorporating body size, the clusters disappear. In their revision of *Raillietiella* taxonomy, Ali et al. [17] state that “body size is not well correlated with hook size, probably because of inconsistencies in fixation combined with allometric growth”, yet we found significant positive correlations in their measurements of AB and BC of both anterior and posterior hooks against body size (their Table 4; *R* ^2^ = 0.57–0.94, *P* = 0.001–0.03). Our quantitative measure of hook bluntness (area of a standardized portion of the hook tip) revealed that larger pentastomes have blunter posterior and anterior hooks. Further, pentastomes from geckos had blunter posterior hooks than did pentastomes from toads. Although bluntness of posterior hooks is generally taken into account when distinguishing between species, we stress the importance of considering body size when scrutinizing all aspects of hook morphology.

The relationship between AB of posterior and anterior hooks changed with body length, but did so differently in the two host species. The AB length of hooks increased faster with somatic growth of *R. frenatus* in toads than it did in *R. frenatus* in geckos, in both sexes. Overall, female pentastomes had larger posterior hooks than males, relative to their body size. Hook size increases following each molt [31], suggesting that data on hook morphology can only be compared between fully adult specimens that have undergone their final molt [14]. Unfortunately, this criterion raises the difficult question of what constitutes a fully adult specimen, and how to discern whether the final molt has taken place. Estimating the proportion of uterine eggs that contain fully developed primary larvae has been proposed as a way to assess maturity in females [3], with females thought to be past the final molt if 20% or more of their eggs contain fully developed primary larvae. Considering body size when scrutinizing differences in morphology may eliminate the need to estimate the percentage of mature uterine eggs, a process that is difficult in small raillietiellids where eggs number ∼5000–9000 [3], and impossible in porocephalids where a single female can have millions of eggs [2].

Morphology of the copulatory spicules is an important feature for species recognition, particularly between raillietiellid taxa where spicules differ in size, shape and ornamentation of the base [14]. The size and appearance of the copulatory spicules of our largest specimens did not differ between *R. frenatus* of toads and those of geckos, and strongly resemble those depicted in Plate 2c of Ali and Riley [3]. Our measurements of the smaller specimens were also grossly comparable with the copulatory spicule measurements reported for pentastomes identified as *R. indica* in *R. marina* of Hawaii, although the larger spicules exceed the upper limit given in that account, our biggest male pentastomes were also larger than those reported in that study [5].

Our morphological data strengthened the molecular-based conclusion in four ways: (1) when pentastome body size was taken into account there was no clustering of hook measurements; (2) hooks became larger and blunter as pentastome body size increased; (3) there were no significant differences between our morphological data and that presented in the literature for both *R. indica* [5] and *R. frenatus* [17]; (4) the pentastomid which retained the posterior pair of hooks from two previous instars demonstrated that the morphology of the distal extremities of the posterior hooks changes from sharply pointed to bluntly pointed to very blunt-ended, the former characteristic of *R. indica* and the latter characteristic of adult *R. frenatus*.

We found *Raillietiella orientalis* in two cane toads. This species was previously known only from Asian snakes [32], so the adult specimens that we recovered from *R. marina* are the first adult *R. orientalis* reported from bufonids, and the first records of this pentastomid taxon in Australia. Previous records of immature *R. orientalis* in bufonids have been interpreted to mean that toads were either an intermediate or an accidental host [32]. *Raillietiella orientalis* infects an Australian elapid (*Demansia vestigiata*) in the vicinity of our study sites (CK, unpublished data) and these pentastomes may pass to toads feeding on snake carrion in the wild. On two occasions we have observed toads feeding on road-killed *Demansia* sp. carcasses locally, perhaps attracted by fly larvae. Pentastomes are known to crawl out of dead hosts, and toads may ingest these snake parasites as they do so. Transplant experiments have demonstrated the capacity for reptilian pentastomes to infect novel bufonid hosts following manual translocation into the glottis [33], thus, toads may occasionally be accidental hosts to ingested adult snake parasites.

Our study clearly shows that the morphological features used in pentastomid taxonomy change as the parasite transitions through different developmental stages in the definitive host and in particular, that the morphology of the hooks changes markedly and progressively. Much work remains to be done in resolving speciation issues in the pentastomid genus *Raillietiella.* We stress that future taxonomic work should involve a combination of morphological techniques, incorporating a consideration of body size and a quantitative measure of hook bluntness, and molecular techniques to assist in the validation of descriptions of new pentastome species. Unfortunately, type specimens in museums are generally fixed in permanent mounts and cannot be characterized by molecular means, a major stumbling block in resolving the taxonomic status of described species of the genus *Raillietiella*.

## Methods

### Ethics Statement

This study was carried out in strict accordance with the recommendations in the Australian Code of Practice for Care and Use of Animals for Scientific Purposes of the National Health and Medical Research Council. This study was approved by the University of Sydney Animal Ethics Committee (L04/4-2008/2/4788; L04/5-2010/2/5334). Extreme care was taken to minimize suffering.

### The Hosts: *Rhinella marina* and *Hemidactylus frenatus*

Native to South and Central America, the cane toad *R. marina* was introduced to Queensland in 1935, and has since spread to cover much of tropical Australia. Initial reports inferred that the original founder toads introduced to Australia were devoid of their native parasite fauna due to numerous successive translocations (Guyana>Barbados>Puerto Rico>Hawaii>Australia) [34]. However, recent studies reveal at least one parasite, a lung nematode (*Rhabdias pseudosphaerocephala*) native to South America infects Australian cane toads and presumably survived these translocations, despite being absent from Hawaiian toad populations [35].

Native to tropical Asia and the Indo-Pacific [36], the gecko *H. frenatus* has naturalized in ∼30 countries [37], its spread aided by human travel and trade. *Hemidactylus frenatus* became established in Darwin in the 1960s, has since spread widely through northern Australia [38] and is infected with a native-range pentastome *R. frenatus* [12].

### Collecting Hosts

Animals were collected by hand from the Darwin Royal Australian Air Force (RAAF) Base golf course (12°25′S, 130°51′E). Toads were collected over the period November 2008–October 2010, and geckos were collected in September 2010. In the course of ongoing surveys within our lab, unusually large pentastomes were found inhabiting the lungs of two toads. These pentastomes were incorporated into the current study – one toad was a male collected in January 2009 from Windows on the Wetland (12°35′S, 131°19′E) and the other, a female toad collected in July 2010 from the Adelaide River township (13°24′S, 131°11′E). Additionally, pentastomes from the lungs of a road-killed female green tree frog (*Litoria caerulea*) collected in May 2011 from The University of Sydney Tropical Ecology Research Facility (12°34′S, 131°18′E) were inspected. All living animals were euthanized with an overdose of pentobarbitone sodium, their lungs were removed, and all pentastomes were collected and immersed alive into cold ethanol (70%).

### Pentastome DNA Extraction and Amplification

For molecular analyses, we processed 26 pentastomes from 11 toads and eight pentastomes from three geckos. In addition, two pentastomes (*Waddycephalus* sp.) from the lungs of two road-killed colubrid snakes (*Stegonotus cucullatus*) were analyzed and used as outgroups. These snakes were collected in November 2008 and April 2010 on local roads surrounding the Research Facility. Ethanol preserved samples were placed in 200 *µ*L of 5% Chelex containing 0.2 mg/mL of proteinase K, incubated overnight at 56°C, boiled at 100°C for 10 min, and centrifuged at 13 300 g for 10 min. The supernatant, containing purified DNA, was removed and stored at −20°C. Double-stranded DNA amplifications of cytochrome c oxydase subunit 1 (cox1) were performed with the primer pair LCO1490 (5′-ggtcaacaaatcataaagatattgg-3′)/HCO2189 (5′-taaacttcagggtgaccaaaaaatca-3′) [39]. Amplification conditions included a hot start denaturation of 95°C for 3 min, followed by 40 cycles of 95°C for 60 s, 55°C annealing temperature for 60 s, 72°C for 105 s, and a final extension of 72°C for 7 min. Sequence reactions were visualized on a 3730xl DNA Analyzer (Applied Biosystems, CA, USA) and aligned using BioEdit [40] and by eye.

### Pentastome Morphology

For morphological observations 26 pentastomes from eight toads, 14 pentastomes from five geckos, and two pentastomes from one tree frog, were cleared in lactophenol, mounted on slides and cover-slipped (small specimens), or flattened with another slide (large specimens), for photographing. Specimens were photographed at 7× for body size measurements using a microscope camera (DCM300, Oplenic) coupled to a dissecting scope (XTL3400D, ProSciTech, Australia), and at 100× for hook and copulatory spicule measurements using the same microscope camera coupled to a compound scope (CX31, Olympus, Australia). Measurements were taken from photographs using ImageJ [41].

**Figure 7:**
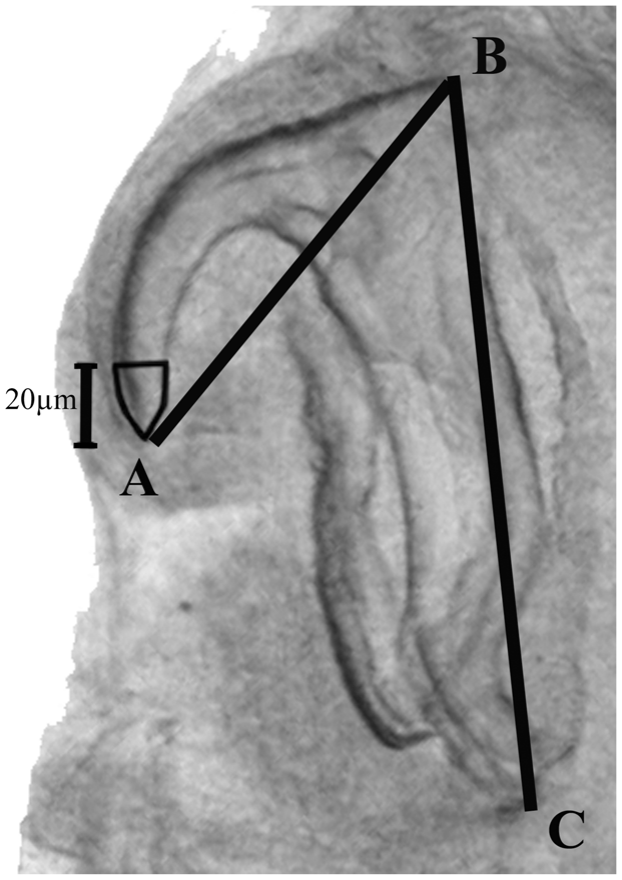
Protocol for measuring pentastome hooks. Photograph of right posterior hook of *Raillietiella frenatus* demonstrating standard procedure for measuring barb length (AB) and overall length (BC), and a new procedure for measuring bluntness of hooks.

All measurements were made with the specimen oriented in ventral view. All specimens measured were either males with formed copulatory spicules or females containing eggs. We did not attempt to count the proportion of uterine eggs contain fully developed primary larvae [3]. Body length was measured from the tip of the head to the end of the caudal segment; body width was measured at the widest point. Hook measurements followed the protocol outlined in Ali et al. [17]. Hook measurements offer consistent morphological features because they are highly sclerotized and are not altered by fixation [14]. Hook dimensions were measured as distances from the hook tip to the inside corner of anterior fulcrum (AB: barb length), and from the back corner of the anterior fulcrum to the outside corner of the posterior fulcrum (BC: overall length; Fig. 7). Plotting AB against BC of posterior hooks is used to visualize distinct clusters, each of which is then considered a separate species [14]. We devised an index to describe bluntness of hooks by measuring the area of a standardized portion of the hook tip, a large area being indicative of a blunt hook, and a small area being indicative of a sharp hook. The area of the tip of each hook was calculated as a polygon created by tracing the perimeter of the hook (using ImageJ [41]) from the tip to 20 *µ*m along the hook shaft (straight-line distance through the centre of the hook shaft; Fig. 7). Length of copulatory spicules was measured by tracing the centre-line of the spicule from start to base; width of copulatory spicules was measured at the widest point of the base. Where possible we measured all four hooks and both copulatory spicules.

Where morphological data is presented from the literature we present only values given for individual specimens where the sex and body size are known. We extracted morphological data from the literature for *R. frenatus* from *H. frenatus* collected in Malaysia, Philippines, Taiwan and Vietnam (from Table 4 in Ali et al. [17]), and female *R. indica* from *R. marina* collected in Hawaii (from Table 1 in Barton and Riley [5]). All specimens were preserved in 70% ethanol, enabling direct comparison of body sizes between studies.

### Data Analyses

All analyses were performed using JMP® 7.0 [42] with alpha set at <0.05. Analyses were performed on mean values for all measurements taken on paired structures (anterior hooks, posterior hooks, and copulatory spicules). For each aspect of hook morphology (AB, BC, area of hook tip) we performed full factorial analyses with pentastome body size, sex and host as independent variables. We tested for significant interactions by sequentially removing the highest order interactions. For comparisons between morphological measurements given in the literature and those presented in the current study, we ran multiple regressions with AB and BC of anterior and posterior hooks as the dependent variables, and pentastome body length and study (Ali et al. [17]; Barton and Riley [5]; Kelehear et al. current study) as the independent variables.
